# Management of displaced radial neck fractures in children: percutaneous pinning vs. elastic stable intramedullary nailing

**DOI:** 10.1007/s10195-013-0252-0

**Published:** 2013-07-11

**Authors:** Luigi Tarallo, Raffaele Mugnai, Francesco Fiacchi, Francesco Capra, Fabio Catani

**Affiliations:** 1 Orthopaedics and Traumatology Department, Modena Policlinic, University of Modena and Reggio Emilia, Via del Pozzo 71, 41124 Modena, Italy; 2 University of Bologna, Bologna, Italy

**Keywords:** Radial neck, Fracture, Metaizeau, ESIN, Closed reduction, Pin, Mayo, Complications

## Abstract

**Background:**

The treatment of radial neck fractures in children varies according to the displacement, angulation, and skeletal maturity. There is a general agreement that displaced radial neck fractures with more than 30° angulations (Judet type III and IV fractures) should be surgically treated. There are several treatment possibilities for Judet type III and IV fractures including percutaneous pin reduction, elastic stable intramedullary nailing (ESIN), and open reduction with or without internal fixation. In this retrospective study we compared the clinical and radiographical outcomes, and complications following intramedullary versus percutaneous pinning in displaced radial neck fractures in children.

**Materials and methods:**

Between 2000 and 2011, 20 patients were treated using closed reduction: in 12 cases we used percutaneous pinning, and in 8 cases we used ESIN. According to Judet classification the two groups were composed as follows: 10 (77 %) type III and 3 (23 %) type IV fractures in the percutaneous pinning group; 4 (57 %) type III, and 3 (43 %) type IV fractures in the ESIN group.

**Results:**

After an average of 42 months, excellent results in Mayo elbow performance scores (MEPS) were obtained in 71 and 69 % of ESIN and percutaneous pinning groups respectively, with good results in the remaining cases apart from one fair case (8 %) in the percutaneous pinning group. After a radiological evaluation, all fractures healed in excellent or good alignment. When comparing the two groups, the subjects treated with the ESIN technique had higher range of motion (ROM) in flexion, extension and pronation. No patients developed complications, except three cases of asymptomatic enlargements of the radial head, reported only in the percutaneous pinning group.

**Conclusion:**

In this research the clinical outcome, assessed with the MEPS, and the radiological alignment, were comparable between the subjects that were treated with percutaneous pinning and those with ESIN techniques; whereas the ESIN technique demonstrated higher ROM in flexion, extension and pronation. The ESIN technique seems to be the ideal approach both for the higher ROM values and for the absence of complications.

## Introduction

The treatment of radial neck fractures in children varies according to the displacement, angulation, and skeletal maturity. Most fractures are undisplaced or minimally displaced (Judet type I and II fractures) and can be treated with closed reduction and casting with good outcome [1]. However, there is a general agreement that displaced radial neck fractures with more than 30° angulations (Judet type III and IV fractures) should be surgically treated [2–4]. There are several treatment possibilities for Judet type III and IV fractures, including percutaneous pin reduction [5], elastic stable intramedullary nailing (ESIN) [6], and open reduction with or without internal fixation [7]. Although open reduction is a method of treatment often used in the past, it is used today in comminuted fractures and cases where closed reduction has failed. In fact, in the literature higher rates of complications are reported after open compared to closed reduction, in particular regarding avascular necrosis (19 vs. 5 %) [8, 9]; premature epiphyseal fusion (50 vs. 5 %) [8, 9], and heterotopic ossifications (25 vs. 4 %) [8, 10].

The proximal radial epiphysis is mainly supplied by periosteal blood vessels running from distal to proximal; the fracture itself or dissection required for open reduction may disturb the blood supply and may lead to avascular necrosis of the radial head or physeal closure [11, 12]. The ESIN technique, as proposed by Metaizeau et al. [13], consists of introducing a pin into the medullary canal of the radius and pushing it proximally until its point reaches the inferior aspect of the epiphysis, lifting it up. This permits extracapsular but intramedullary reduction and fixation combining closed reduction and minimal invasive internal fixation with preservation of the soft-tissue attachments [14]. The present study is a retrospective analysis comparing the clinical and radiographical outcomes and complications following intramedullary versus percutaneous pinning in a consecutive cohort of children with displaced radial neck fracture.

## Materials and methods

### Patients

All patients and their parents gave informed consent prior to being included in the study. This retrospective research was approved by our institutional ethics committee and was performed in accordance with the ethical standards of the 1964 Declaration of Helsinki as revised in 2000. Inclusion criteria were: a recent closed, displaced radial neck fracture with an angulation of more than 30° (Judet type III and IV) in children with open growth plates (<16 years). Between 2000 and 2011, 21 children, with ages ranging between 6 and 16 years, with an average of 11 years, were treated in our university hospital. According to Judet classification, there were 15 (73 %) type III, and 6 (27 %) type IV fractures. Twelve (59 %) patients were boys and nine (41 %) were girls. The dominant side was injured in 16 (76 %) patients. Two patients had an associated compound fracture of the ipsilateral olecranon, and one patient had displaced olecranon fracture with a lesion of the lateral collateral ligament (LCL). The two cases with an associated compound olecranon fracture were treated conservatively, whereas the case with a displaced olecranon fracture with a lesion of the LCL was surgically treated with closed Kirschner wire-assisted reduction.

### Surgical techniques

Twenty patients were treated using closed reduction: in 12 cases we used percutaneous pinning, and in the remaining eight cases we used ESIN. The choice of the surgical technique (percutaneous or ESIN) depended on the surgeon’s abilities and preferences. Open reduction was necessary only in one case, where alignment with manual and closed reduction was not possible to achieve. Intraoperatively in these cases, soft tissue interposition, which had hindered reduction during the operation, was identified, and stabilization was provided with the help of an intramedullary K-wire by conducting open reduction with minimal arthrotomy. When the child was under general anesthesia, using an image intensifier, we first attempted to reduce the displaced fracture with pressure on the lateral side with longitudinal traction and varus stress with the elbow extended. Extremely forcible manipulations for achieving ideal reduction were avoided.

### Elastic stable intramedullary nailing

A K-wire was contoured and bent at the tip at an angle of 30–45°. Depending on the age of the child, the diameter of the pin used was 1.2–2.0 mm. The distal radial epiphysis of the radius was identified using an image intensifier and a longitudinal 0.5-cm postero-radial skin incision overlying the distal radial metaphysis was made. The soft tissue was dissected by taking care not to injure the cutaneous branch of the radial nerve. The lateral cortex was exposed and perforated using a Pfriem-type trocar and the curved flexible pin was inserted and pushed cranially until it reached the inferior aspect of the displaced epiphysis. At this stage, the intramedullary wire was gentle pushed so that the point fixed in the epiphysis, in order to raise it up to reposition under the lateral condyle. In order to reduce the remaining radial-lateral displacement, the wire was rotated through 180° so that the tip pointed towards the ulna, thereby forcing the fragment to shift medially and facilitating anatomical reduction of the radial head within the joint. The distal part of the pin was cut and bent, and the skin was closed. If the reduction was still not satisfactory, a K-wire was inserted percutaneously, through the fracture from the lateral side and used as a lever arm to reduce the fracture, as suggested by Bohler [15]. This association of the Metaizeau and Bohler techniques was necessary in three cases.

**Fig. 1:**
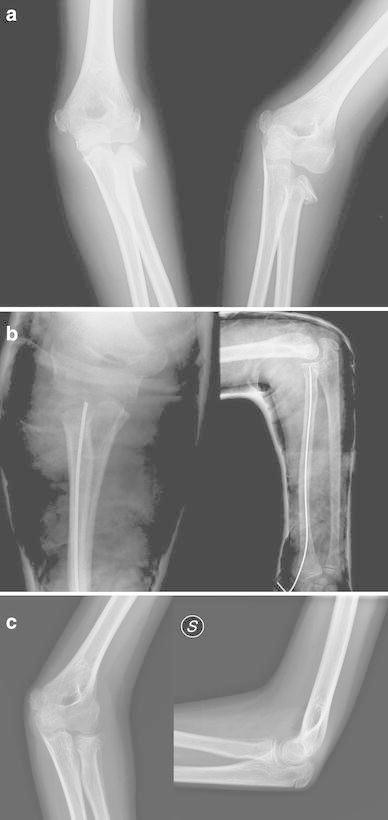
**a** Radial neck fracture Judet type III. **b** Osteosynthesis with ESIN, postoperative X-rays. **c** X-rays at 3 months, after nail removal, showing an excellent alignment

A long-arm cast with the forearm in a neutral position was applied and maintained until K-wire removal, performed 3–4 weeks after surgery (Fig. 1). After K-wire removal, a volar long-arm cast was applied for further 30 days. Therefore, arm mobilization and exercising started 2 months after surgery, at the removal of the volar cast.

### Percutaneous pinning

**Fig. 2:**
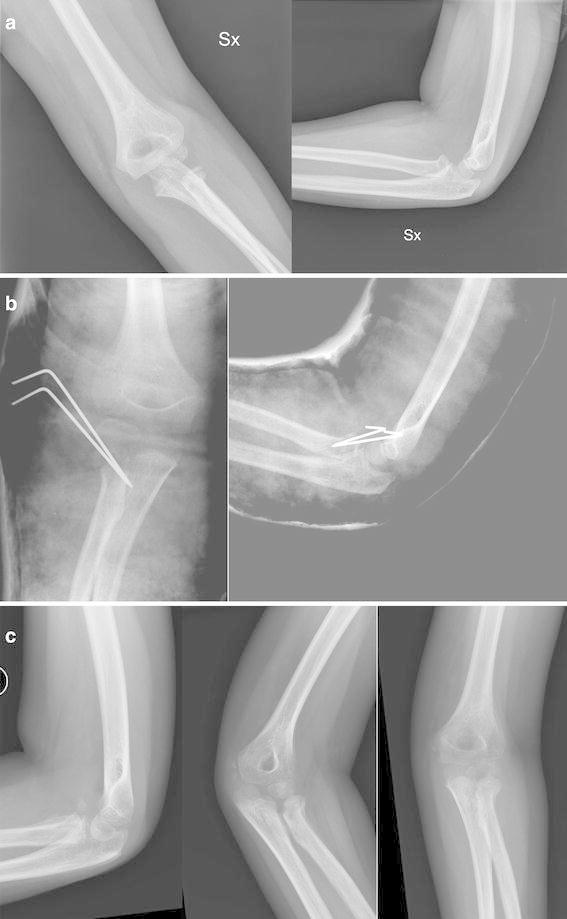
**a** Radial neck fracture Judet type III. **b** Osteosynthesis with two percutaneous K-wires, postoperative X-rays. **c** X-rays at 3 months, after K-wires removal, showing a good alignment

A 1.4- to 1.6-mm K-wire was used, depending on patient age. The K-wire was inserted percutaneously from proximal to distal radial neck fracture site, the outside part of the K-wire was pulled distally through the skin and the inner side of the K-wire was then inserted around the fracture site. In this procedure, care must be taken to avoid penetration of the K-wire into the fracture site. As in the Kapandji [16] technique for treating fractures of the distal radius, the inserted K-wire was used as a lever, the radial neck fracture was reduced and reduction was confirmed with an image intensifier. After reduction, the K-wire was advanced toward the ulnar side to impact the opposite cortex. After leverage of the displaced radial head or fragment, the wire was advanced to impact the opposite cortex. In seven of the 12 cases, an additional K-wire was introduced in the same way. The stability of reduction and forearm rotation were checked under fluoroscopic control. The K-wire was left protruding out of the skin and was bent over to prevent migration. A long-arm cast with the forearm in a neutral position was applied (Fig. 2). The post-operative procedure was the same as for the patients treated with the ESIN technique, previously reported.

### Outcomes

It’s our routine practice to evaluate clinically and radiologically all patients at 1 month, at 2 or 3 months, and thereafter at 6-monthly intervals. After obtaining informed consent from patients or patientsʼ parents, all radiographs and hospital records were reviewed; moreover, all 20 patients treated with closed reduction received an extra clinical follow-up between May and June 2012. The follow-up period ranged from 15 to 63 months, with a mean of 42 months. The angulation of the radial neck was measured as the angle between a line drawn parallel to the superior articular surface of the radial head and a line perpendicular to the articular surface through the radial shaft in the primary radiographs. The postoperative clinical evaluation was performed by one of the authors (F.F.) and included analysis of passive and active range of motion (ROM), radiological evaluation of alignment, functional results using the Mayo elbow performance score (MEPS), and early or late complications. Flexion and extension of elbows, pronation and supination of the forearm and the angle of the extended elbows were measured by a goniometer. The uninjured elbows served as controls. The last follow-up radiographs included standard anteroposterior and lateral projections of the injured elbow. All measurements were performed on a picture archiving and communication system (PACS, software Fuji Synapse). Radiologically, the reduction was considered excellent when it healed in the anatomical position; good when the radial neck angle was less than 20°; medium when the angle was between 20° and 40°; poor with an angle of more than 40°. The MEPS is one of the most commonly used physician-based elbow rating systems (Table 1). The joint’s stability was graded as stable, mildly unstable or unstable. The functional score is determined on the basis of the patient’s ability to perform normal activities of daily living. The total score ranges from 5 to 100 points, with higher scores indicating better function. If the total score is between 90 and 100 points, it can be considered excellent; between 75 and 89 points, good; between 60 and 74 points, fair; less than 60 points, poor [17]. We decided to use the MEPS as it can be completed quickly, it assesses elbow’s function and pain by questions, and the condition of the elbow by objectively measured clinical data and all its items are applicable in pediatric subjects. Possible complications such as avascular necrosis, nonunion, proximal synostosis, heterotopic ossification, infection, and premature physeal closure were documented.

**Table 1:** Mayo elbow performance score (MEPS)

Variable	Definition	No. of points
Pain (max., 45 points)	None	45
	Mild	30
	Moderate	15
	Severe	0
Range of motion (max., 20 points)	Arc >100°	20
	Arc 50–100°	15
	Arc <50°	5
Stability (max., 10 points)	Stable	10
	Moderately unstable	5
	Grossly unstable	0
Function (max., 25 points)	Able to comb hair	5
	Able to feed oneself	5
	Able to perform personal hygiene tasks	5
	Able to put on shirt	5
	Able to put on shoes	5

### Statistical analysis

In the case of normal distribution, an independent samples *t*-test was used to define the two-sided probability of statistical significance. In the analyses that reported an *F* test *p* value of less than 0.05 (variances of the two samples cannot be assumed to be equal) a *t-*test with correction for unequal variances (Welch test) was applied. When the distribution was nonparametric, a Mann–Whitney test for independent samples was performed. Statistical analyses were performed using MedCalc for Windows, version 12.2.1 (MedCalc Software, Mariakerke, Belgium).

## Results

At an average 42-month follow-up, all 20 patients treated with ESIN or percutaneous pinning techniques were clinically and radiographically evaluated. In Table 2 we report the MEPS score and radiological evaluation. When examining the two groups, excellent results in the MEPS score were obtained in 71 and 69 % of the ESIN and percutaneous pinning groups respectively, with good results in the remaining cases, apart from one fair case (8 %) in the percutaneous pinning group, represented by the patient with an associated displaced olecranon fracture with a lesion of the LCL (Table 2).

**Table 2:** Clinical and radiological comparison between the ESIN and percutaneous pinning group

	Associated injuries	MEPS				Radiological alignment	
		Excellent *N* (%)	Good *N* (%)	Medium *N* (%)	Poor *N* (%)	Excellent *N* (%)	Good *N* (%)
ESIN							
All fractures (*N* = 7)		5 (71)	2 (29)	–	–	6 (67)	1 (33)
Type III (*N* = 4)		3 (75)	1 (25)	–	–	4 (100)	–
Type IV (*N* = 3)	1 Olecranon fracture (*N* = 1)	2 (67)	1 (33)	–	–	2 (67)	1 (33)
Percutaneous pinning							
All fractures (*N* = 13)		9 (69)	3 (23)	1 (8)	–	10 (77)	3 (23)
Type III (*N* = 10)	1 Olecranon fracture (*N* = 1)	8 (80)	2 (20)	–	–	9 (90)	1 (10)
Type IV (*N* = 3)	Olecranon fracture + LCL lesion (*N* = 1)	1 (33)	1 (33)	1 (33)	–	1 (33)	2 (67)

Radiological alignment: *excellent*, anatomical reduction; *good*, angle less than 20°; *medium*, angle of 20°–40°; *poor*, angle of more than 40°

*LCL* lateral collateral ligament

Radiological evaluation revealed that all fractures healed in excellent or good alignment. In particular, an excellent alignment was reported in 67 and 77 %, of ESIN and percutaneous pinning groups, respectively. It can be observed that higher degrees of fracture displacement determined poorer postoperative radiological alignment (Table 2). The ROM evaluation is reported in Table 3.

**Table 3:** Range of motion comparison between ESIN and percutaneous pinning groups

	Flexion (°) (mean ± SD)		Extension (°) (mean ± SD)		Pronation (°) (mean ± SD)		Supination (°) (mean ± SD)	
	Fractured	Uninjured	Fractured	Uninjured	Fractured	Uninjured	Fractured	Uninjured
ESIN (*N* = 7)								
All fractures	145 ± 6	152 ± 5	1 ± 1	0	85 ± 4	85 ± 3	86 ± 3	86 ± 3
Type III	148	150	0	0	90	90	85	85
Type IV	146 ± 6	151 ± 5	1 ± 1	0	85 ± 5	85 ± 3	86 ± 3	86 ± 3
Percutaneous pinning (*N* = 13)								
All fractures	140 ± 14	151 ± 4	2 ± 3	0	83 ± 7	85 ± 2	82 ± 12	85 ± 3
Type III	115 ± 28	152 ± 6	6 ± 5	0	73 ± 18	86 ± 1	62 ± 31	82 ± 3
Type IV	139 ± 5	154 ± 2	2 ± 2	0	82 ± 2	84 ± 2	85 ± 3	85 ± 3

When comparing the two groups, the subjects treated with the ESIN technique had higher ROM in flexion, extension and pronation (Table 4).

**Table 4:** Groups comparison and statistical analysis

	MEPS	Radiological alignment (°)	Range of motion (°)			
			∆ flexion	∆ extension	∆ pronation	∆ supination
ESIN (*N* = 7)	91.00 ± 7.30	1.43 ± 3.78	3.00 ± 2.08	0.29 ± 0.76	0.00 ± 0.00	0.00 ± 0.00
Percutaneous pinning (*N* = 13)	91.08 ± 10.74	2.69 ± 5.25	31.92 ± 28.03	5.00 ± 4.56	11.92 ± 14.94	18.46 ± 29.32
ESIN vs. percutaneous pinning	0.9867^b^	0.6391^c^	*0.0030* ^*a*^	*0.0135* ^*c*^	*0.0151* ^*c*^	0.1448^c^

Data expressed as mean ± standard deviation

∆ difference in range of motion between uninjured and fractured arm

^a^Welch test

^b^*t*-test

^c^Mann–Whitney test

No patients developed nonunion, avascular necrosis, infection, periarticular ossification, radioulnar synostosis, nerve injury, or premature physeal closure. The only “minor” complication observed was represented by three cases of asymptomatic enlargements of the radial head, reported only in the percutaneous pinning group.

## Discussion

This study was conducted to compare the clinical and radiographical outcomes, and complications rate in patients with displaced radial neck fractures treated with two closed reduction methods: ESIN and percutaneous pinning techniques. We agree with the majority of authors who recommend treating fractures with an angulation of less than 30° (Judet type I and II fractures) conservatively and treating those with an angulation more than 30° (Judet type III and IV) surgically [2, 6, 7, 18]. There are several treatment possibilities for Judet type III and IV fractures including: percutaneous pinning [5], ESIN technique proposed by Metaizeau et al. [6, 19], and open reduction with or without internal fixation [7]. It is widely reported in the literature that open reduction leads to worse outcomes instead of a good reduction of the fracture [5, 20–22]. In fact, after open reduction, the incidence of avascular necrosis, proximal synostosis, heterotopic ossification, infection, premature physeal closure, and loss of ROM is higher than after closed reduction [8, 20, 22, 23]. Therefore, percutaneous methods of reduction have been developed in an effort to avoid the higher incidence of complications associated with open reduction. However, some radial neck fractures, in particular severely displaced, are impossible to reduce with closed methods, requiring open reduction [7, 24, 25]. Open reduction is inevitable in cases of comminuted fractures, interposition of the capsule or anular ligament between the head and the neck, totally displaced, and fracture dislocation [26].

In recent decades, newer methods of treatment have been proposed to treat these difficult, severely displaced fractures. The intramedullary fixation technique described by Metaizeau et al. [13] in 1980 achieves acceptable indirect reduction with the preservation of the lateral periosteum and the epiphyseal vascular supply, associated with internal fixation that prevents displacement during the healing period. In 1993 he further developed this method to reduce and fix the displaced radial neck fracture by the use of elastic stable intramedullary nails [14].

Several authors reported good results using this technique [9, 27, 28]; however, we know of no previous studies reported in the literature comparing the clinical and radiographical outcomes, and complications, following intramedullary versus percutaneous pinning techniques. In this research, the clinical outcome, assessed with the MEPS, and the radiological alignment were comparable between the subjects that were treated with percutaneous pinning or ESIN techniques. The ROM evaluation revealed higher degrees in flexion, extension and pronation in the subjects treated with the ESIN technique. No patients developed nonunion, avascular necrosis, infection, periarticular ossification, radioulnar synostosis, nerve injury, or premature physeal closure. The only observed “minor” complication was represented by three cases of asymptomatic enlargements of the radial head, reported only in the percutaneous pinning group. In our study, open reduction was necessary only in one case, where adequate reduction was hindered by interposition of the anular ligament. Our findings are in line with a similar study of Klitscher et al. [29] that reported excellent results in 82 % and good in 5 % of cases, using the MEPS score. This is the first study that compares two different closed reduction and fixation methods for the treatment of displaced radial neck fractures in children. In conclusion, both methods gave excellent clinical and radiological results with few complications. However, based on the results reported in the present study, the ESIN technique seems to be the preferred approach both for the higher ROM values and for the lower rate of complications. Certainly, these results must be confirmed by further studies with higher methodological standards, including randomization, and larger sample size.
